# Root Suberin Forms an Extracellular Barrier That Affects Water Relations and Mineral Nutrition in Arabidopsis

**DOI:** 10.1371/journal.pgen.1000492

**Published:** 2009-05-22

**Authors:** Ivan Baxter, Prashant S. Hosmani, Ana Rus, Brett Lahner, Justin O. Borevitz, Balasubramaniam Muthukumar, Michael V. Mickelbart, Lukas Schreiber, Rochus B. Franke, David E. Salt

**Affiliations:** 1 Bindley Bioscience Center, Purdue University, West Lafayette, Indiana, United States of America; 2 Horticulture and Landscape Architecture, Purdue University, West Lafayette, Indiana, United States of America; 3 Department of Ecology and Evolution, University of Chicago, Chicago, Illinois, United States of America; 4 Institute of Cellular and Molecular Botany, University of Bonn, Bonn, Germany; The University of North Carolina at Chapel Hill, United States of America

## Abstract

Though central to our understanding of how roots perform their vital function of scavenging water and solutes from the soil, no direct genetic evidence currently exists to support the foundational model that suberin acts to form a chemical barrier limiting the extracellular, or apoplastic, transport of water and solutes in plant roots. Using the newly characterized *enhanced suberin1* (*esb1*) mutant, we established a connection in *Arabidopsis thaliana* between suberin in the root and both water movement through the plant and solute accumulation in the shoot. *Esb1* mutants, characterized by increased root suberin, were found to have reduced day time transpiration rates and increased water-use efficiency during their vegetative growth period. Furthermore, these changes in suberin and water transport were associated with decreases in the accumulation of Ca, Mn, and Zn and increases in the accumulation of Na, S, K, As, Se, and Mo in the shoot. Here, we present direct genetic evidence establishing that suberin in the roots plays a critical role in controlling both water and mineral ion uptake and transport to the leaves. The changes observed in the elemental accumulation in leaves are also interpreted as evidence that a significant component of the radial root transport of Ca, Mn, and Zn occurs in the apoplast.

## Author Summary

The root system is a highly specialized plant organ that works to get both water and essential mineral nutrients from the changing chemically and physically complex environment of the soil. Roots do this by both controlling the uptake of water and essential mineral ions, as well as regulating their movement to the central vascular system of the plant for long distance transport to the shoot. To allow the cellular control of water and mineral ion uptake and transport via specialized transport proteins, plant roots contain a waxy layer of suberin that acts to seal connections between cells, preventing uncontrolled leakage of water and mineral ions between cells. By screening thousands of mutant *A. thaliana* plants, we were able to identify a plant with elevated levels of suberin in the root. Using this mutant, we were able to uncover the importance of suberin in sealing connections between root cells to regulate water movement through the plant and accumulation of various essential and nonessential minerals in leaves, including sodium, sulfur, potassium, calcium, manganese, zinc, arsenic, selenium, and molybdenum.

## Introduction

The plant root is a specialized organ that allows uptake of water and selective uptake of solutes from the soil environment, to support normal plant growth and development. To accomplish this function roots take up water and solutes at the root surface and transport them across the root to the xylem vessels in the central vascular tissue in the stele, where they are transported to the shoot. Transport across the root to the central xylem vessels can occur through the extracellular space (apoplastic) or via cell-to-cell transport (symplastic). Specificity of both water and solute transport is generally thought to be provided by transport proteins at the plasma membrane of root cells. However, to achieve such specificity, the non-specific apoplastic transport pathway between cells needs to be regulated. A long held model to explain this regulation includes the function of the extracellular biopolymer suberin acting as a barrier to limit the apoplastic transport of water and solutes. Plant roots contain two such suberin barriers, at the exodermis and the endodermis. Endodermal suberin is thought to prevent the apoplastic movement of water and solutes into the stele, whereas exodermal suberin blocks apoplastic transport at the root surface. Though central to our understanding of how roots function, a careful review of the literature reveals a lack of genetic evidence to support this foundational model.

The effects of altered root suberin content on water relations and ion transport at both the exodermis and endodermis have been tested experimentally by modifying the cultivation conditions or utilizing roots at different developmental stages [Bibr pgen.1000492-Hose1] [reviewed in 1 & 2]. For example, roots of maize grown hydroponically lack a suberized exodermis when compared to aeroponically grown plants [2]. Such differences have been utilized to identify negative correlations between suberin content and root hydraulic conductivity, and radial transport of ABA [1],[3],[4]. Higher suberin content within mature endodermis also results in decreased Ca translocation at different developmental stages along the root of *Cucurbita pepo* [5]. Further, in species with an exodermis and endodermis (*Zea mays*, *Allium cepa*, and *Helianthus annuus*), or just an endodermis (*Vicia faba* and *Pisum sativum*), higher root suberin, in mature regions of the root, correlates with lower apoplastic transport of fluorochromes [6] and root water loss [7]. In concluding that quantitative differences in suberin content, in either the exodermis or endodermis of the root, determines the permeability of the apoplast to both water and solutes the implicit assumption is that differences observed in these physiological parameters are related to the altered suberin contents observed. It has previously been suggested that qualitative differences in the compositions of suberin also need to be considered [8]. Furthermore, given the varied growth conditions, developmental stage or species used in these studies it is quite feasible that differences other than suberin are either directly or indirectly responsible for the observed effects on water relations and ion transport.

Direct evidence for a role of suberin in regulating apoplastic radial transport requires a plant harboring a mutation that specifically alters the amount of suberin deposited in the root. Currently, there are only three reports of *Arabidopsis thaliana* mutants with altered suberin. As it is generally accepted that *A. thaliana* roots do not contain an exodermis [9], suberin in these mutants is likely altered in the endodermis. Knockout alleles of GPAT5 (encoding an acyl-CoA: glycerol-3-phosphate acetyltransferase) in *A. thaliana* were recently shown to contain 20–50% less C_20_–C_24_ aliphatic monomers in root suberin [10]. However, the effect of loss-of-function of GPAT5 on either water relations or ion-transport was not directly measured. The recently isolated *A. thaliana* mutants *horst-1* and *horst-2* containing knockout alleles of the cytochrome P450 fatty acid ω-hydroxylase CYP86A1 gene contain 60% less total aliphatic suberin monomers in roots than wild-type plants [11],[12]. Knockout alleles of the DAISY locus, which encodes a β-ketoacyl-CoA synthase involved in the formation of docosanoic acid, also have altered aliphatic suberin monomer composition, including a decrease in C_22_ and C_24_ fatty acid derivatives and an increase in C_16_, C_18_ and C_20_ fatty acid derivatives [13]. No evidence for impacts on water relations or ion-transport for either the *horst* or *DAISY* mutants was reported.

Given the assumed role of suberin in regulating radial transport of solutes in the root, we would predict that perturbations in suberin content and/or its composition would affect the elemental composition, or ionome, of the shoot. High-throughput screening of the shoot ionome may therefore identify mutants with altered root suberin. We have identified numerous ionomics mutants in a previously reported screen [14], and here we report on the cloning and characterization of one of these ionomics mutants, originally reported as mutant *14501* [14]. Here we report that *14501* results in a doubling of all the aliphatic monomer components of root suberin, and we renamed this mutant *enhanced suberin1* (*esb1-1*). This elevated suberin, most likely in the endodermis given that *A. thaliana* roots lack an exodermis, results in a reduction in transpiration, reduced wilting after water withdrawal, and a perturbation of the shoot ionome, including increases in Na, S, K, As, Se and Mo, and decreases in Ca, Mn and Zn. Grafting experiments confirm that these phenotypes are the result of elevated root suberin. This report provides direct evidence that suberin acts in the root as a barrier to the extracellular transport of both water and mineral ions.

## Results

### Alterations in the Leaf Ionome of *Esb1*

The *A. thaliana* leaf ionomic mutant *14501*, now termed *esb1-1*, was originally reported to have elevated leaf K, Mo and Cd and reduced Ca and Fe compared to wild-type plants [14]. Here we extend this observation and establish with two independent alleles (*esb1-1* and *esb1-2*) that this mutant shows significant (P≤0.01) increases in shoot concentrations of Na, S, K, As, Se and Mo and reductions in Ca, Mn and Zn. We also observed a significant (P≤0.05) reduction in Fe (Table 1). We further note that during earlier experiments characterizing this mutant, with a different batch of the same soil type (in trays 533–535 and 590–594, see www.ionomicshub.org) the shoot concentration of B was also significantly (P≤0.01) reduced by approximately 25–40% compared to wild-type plants. Even though the shoot ionomes of *esb1-1* and *esb1-2* are significantly altered compared to wild-type plants we observed no major phenotypic differences.

**Table 1:** Elemental content of leaf tissue of wild-type and *esb1 A. thaliana* (Col-0).

Elements	Wild-type (WT) *(µg g^−1^ dry weight)*	*esb1-1 (µg g^−1^ dry weight)*	*esb1-2 (µg g^−1^ dry weight)*	*esb1-1 (% diff. from WT)*	*esb1-2 (% diff. from WT)*	*esb1-1 (P values)*	*esb1-2 (P values)*
**Li**	13.11±4.82	11.98±4.28	11.39±3.38	−8.62	−13.18	0.23	0.05
**B**	120.37±21.98	128.05±22.82	115.69±21.11	6.38	−3.89	0.10	0.29
**Na**	**1351.43**±**590.81**	**1907.44**±**1159.52**	**1735.78**±**868.36**	**41.14**	**28.44**	**<0.01**	**0.01**
**Mg**	18787.67±3306.19	17528.63±2205.21	17562.68±2337.85	−6.70	−6.52	0.03	0.04
**P**	8278.83±1502.87	7884.80±1249.43	8534.65±1574.05	−4.76	3.09	0.17	0.42
**S**	**7955.26**±**1023.99**	**9860.71**±**1069.62**	**10514.24**±**1358.69**	**23.95**	**32.17**	**<0.01**	**<0.01**
**K**	**35244.15**±**5430.44**	**45797.41**±**6341.30**	**43599.04**±**7919.22**	**29.94**	**23.71**	**<0.01**	**<0.01**
**Ca**	**34980.61**±**5797.58**	**18092.19**±**2025.49**	**18230.80**±**3516.00**	**−48.28**	**−47.88**	**<0.01**	**<0.01**
**Mn**	**132.94**±**47.34**	**94.28**±**32.55**	**89.17**±**27.00**	**−29.08**	**−32.93**	**<0.01**	**<0.01**
**Fe**	69.69±14.26	63.28±16.06	63.60±14.77	−9.21	−8.74	0.04	0.04
**Co**	1.15±0.44	0.95±0.31	1.04±0.45	−17.40	−9.88	0.01	0.22
**Ni**	6.29±6.81	4.18±4.11	5.77±8.83	−33.57	−8.31	0.07	0.75
**Cu**	1.24±0.51	1.48±0.71	1.36±0.56	20.11	10.29	0.05	0.25
**Zn**	**92.55**±**12.95**	**78.98**±**11.65**	**83.08**±**13.22**	**−14.66**	**−10.23**	**<0.01**	**<0.01**
**As**	**0.23**±**0.14**	**0.52**±**0.31**	**0.47**±**0.18**	**126.92**	**107.20**	**<0.01**	**<0.01**
**Se**	**8.73**±**2.01**	**10.54**±**2.55**	**11.36**±**2.42**	**20.69**	**30.12**	**<0.01**	**<0.01**
**Mo**	**4.38**±**1.88**	**5.84**±**2.91**	**5.82**±**2.90**	**33.17**	**32.85**	**<0.01**	**0.01**
**Cd**	3.62±1.20	3.44±1.12	3.64±1.10	−5.02	0.43	0.44	0.95

For each element data is derived from the analysis of samples from 48 independent plants derived from three separate replicate experiments. Data highlighted in bold represents those elements that are significantly different (t-test P≤0.01) in both alleles of *esb1* compared to wild-type plants. The raw elemental concentrations for individual plant samples are available at www.ionomicshub.org in experimental trays 1095, 1146 and 1279.

### Mapping of the Causal DNA Polymorphism in *Esb1*

**Figure 1:**
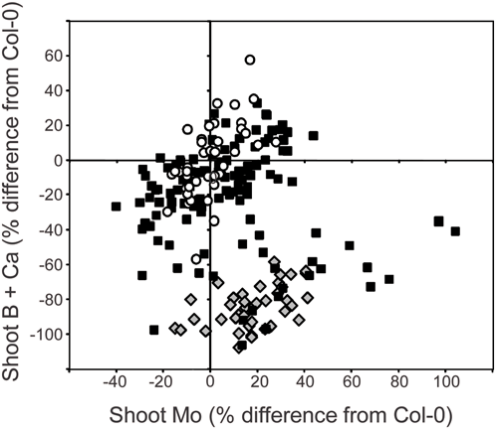
Segregation of shoot ionomic phenotype in five week old F2 plants from a Col-0 x *esb1-1* backcross. Data represents the percentage difference from the mean of the wild-type (Col-0) control plants (n = 40). Open circles = wild-type Col-0; grey diamonds = *esb1-1*; solid squares = F2 plants from a Col-0 x *esb1-1* cross. Raw data can be viewed and obtained from www.ionomichub.org in trays 533, 534 and 535.

Ionomic analysis of leaf tissue sampled from an F2 population derived from the backcross Col-0 x *esb1-1* revealed that 20 F2 plants from a total of 117 showed the mutant ionomic phenotype scored as the percentage difference from wild-type in leaf concentrations of B, Ca and Mo. Figure 1 shows that these mutant F2 plants cluster with the *esb1-1* mutant. The number of F2 plants showing the mutant phenotype is consistent with the hypothesis that the ionomic phenotype of *esb1-1* is caused by a recessive monogenic mutation.

**Figure 2:**
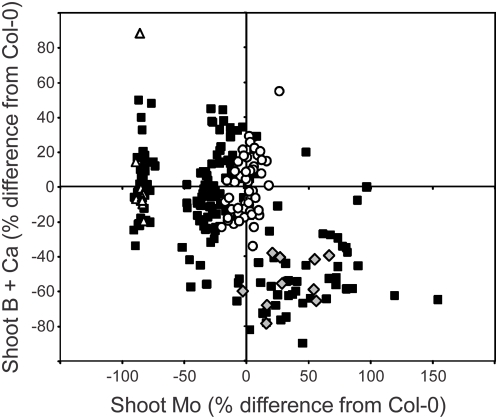
Segregation of shoot ionomic phenotype in five week old F2 plants from a L*er*-0 x *esb1-1* outcross. Data represents the percentage difference from the mean of the wild-type (Col-0) control plants (n = 60). Open circles = wild-type Col-0; grey diamonds = *esb1-1*; open triangles = L*er*-0; solid squares = F2 plants from a L*er*-0 x *esb1-1* outcross. Raw data can be viewed and obtained from www.ionomichub.org in trays 590, 591, 592, 593 and 594. The ∼100% low Mo accumulation of some of the plants is due the effect of the *mot1* ^Ler^ locus [41].

**Figure 3:**
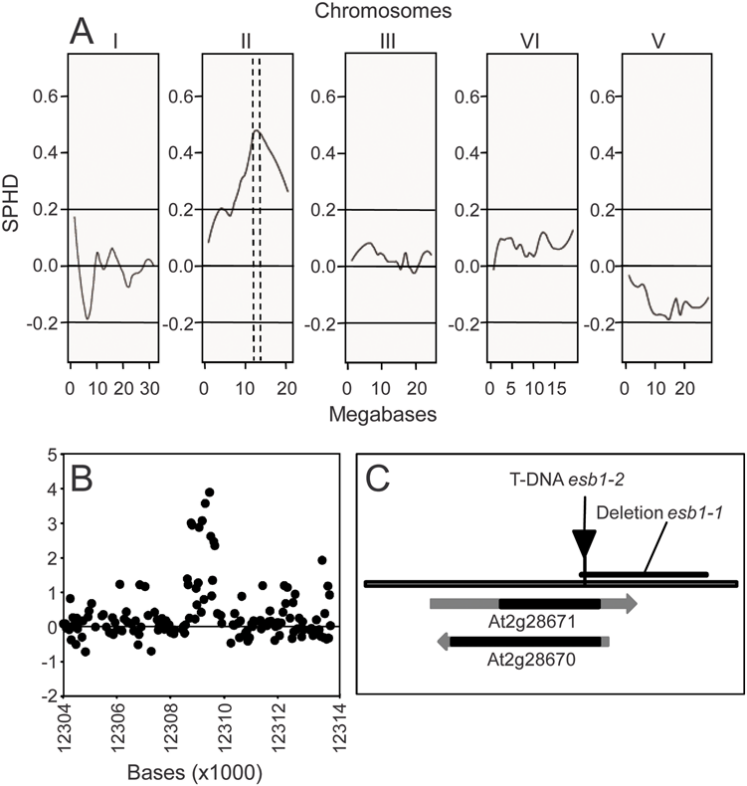
DNA microarray-based BSA, deletion mapping and gene structure of At2g28670. A. Bulk Segregant analysis of the low shoot Ca and B content in an F2 population from a L*er*-0 x *esb1-1* outcross. Data are presented as a scaled pool hybridization difference (SPHD), representing the difference between the hybridization of the two pools at the SFPs, scaled so that a pure Col-0 pool would be at 1 and a pure L*er*-0 pool would be at −1. The pools were prepared from F2 plants with a low Ca and B content (n = 41) and F2 plants with a Col-0-like Ca and B content (n = 41). SFPs were scored after hybridization of genomic DNA prepared from these pools to Affymetrix ATH1 DNA microarrays. Dotted lines denote likely location of the causal loci. B. Deletion analysis of *esb1-1*. DNA from *esb1-1* and wild-type Col-0 was hybridized to the Affymetrix Arabidopsis ATTILE 1.0R microarray and compared the hybridization at probes which represent sequence between 9 and 13 Mb on chromosome 2. C. Grey line represents chromosome 2 between 12,307,000 and 12,310,000 and shows the open reading frames for At2g28671 and At2g28670 (grey arrow representing the cDNA and the black segment representing the single exon). The black triangle represents the T-DNA insertion at 12,308,657 in line *esb1-2* (GABI_858D03), and the solid black line represents the deletion in *esb1-1*.

To map the causal locus of *esb1-1* an outcross to *A. thaliana* L*er*-0 was made and the ionomic phenotype of 175 F2 plants determined, and represented as the percentage difference from wild-type Col-0 for B, Ca and Mo (Figure 2). To obtain a rough map position a bulk segregant analysis (BSA) experiment [15] was performed with microarray detection of genetic markers [16],[17], using (*esb1-1* x L*er*-0) F2 plants. Plants with the lowest Ca and B content (n = 41), that clustered with *esb1-1*, and plants with B and Ca shoot contents similar to Col-0 (n = 41) were pooled separately, and genomic DNA from each pool hybridized to the Affymetrix Arabidopsis ATH1 microarray. Using the oligonucleotide probes on the DNA microarray that show differential hybridization between L*er*-0 and Col-0 as genetic markers (Single Feature Polymorphisms or SFP), the locus responsible for the ionomics phenotype in *esb1-1* was mapped to an area centered at 11 Mb on chromosome 2 (Figure 3A). To identify the causal DNA polymorphism in *esb1-1* we hybridized DNA from *esb1-1* and wild-type Col-0 to the Affymetrix Arabidopsis ATTILE 1.0R microarray and compared the hybridization at probes that represent sequence between 9 and 13 Mb on chromosome 2 (Figure 3B). Nineteen probes covering the region from 12,308,779 to 12,309,878 showed log2 intensity differences ranging from 0.23 to 3.57 with an average difference of 1.8. Sequencing of this region revealed a 1097 base pair deletion starting at 12,308,627 and ending at 12,309,723. This deletion lies within a genomic region containing the 2 putative open reading frames (ORFs) At2g28670 and At2g28671 (Figure 3C), neither of which are predicted to contain introns. The deletion is within the predicted promoter region of At2g28670 and the 3′ end of At2g28671. *Esb1-2* (GABI_858D03) has a T-DNA insertion at 12,308,657. We are aware that At2g28670 has been previously called *DIR10* [18]. However, this naming is not based on any published functional evidence, but rather unpublished phylogenetic data. At2g28670 is annotated as a “disease resistance-responsive family protein/fibroin-related” gene, however there are currently no published studies describing the function of this gene. The second predicted ORF in this genomic region At2g28671 has been recently automatically annotated at The Arabidopsis Information Resource (TAIR) (www.arabidopsis.org) as encoding a protein containing the signature prichextensn domain of proline-rich extensins, based on similarity to the maize extensin protein [19]. However, none of the 54 ESTs reported in TAIR (www.arabidopsis.org) from this genomic region are consistent with expression of At2g28671, but rather they all support expression of At2g28670. Furthermore, no promoter is predicted for the putative At2g28671 ORF using TSSP [20], whereas a strong promoter is predicted for At2g28670. We therefore concluded that At2g28670 is the most likely functional gene at this locus.

**Figure 4:**
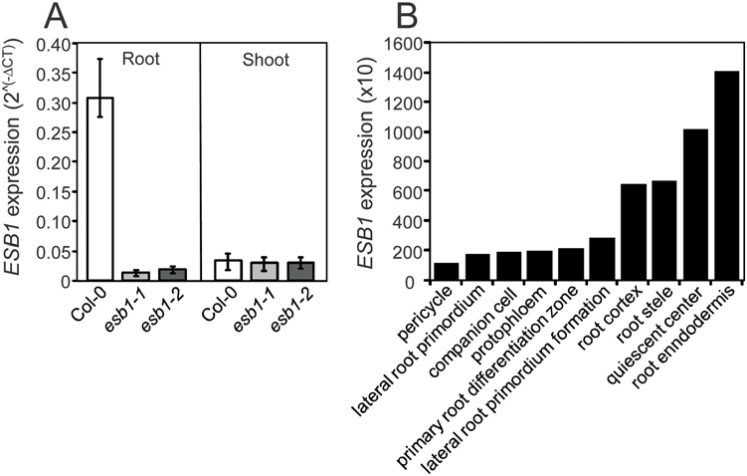
Expression pattern of At2g28670. A. Expression of At2g28670 was analyzed by quantitative real-time RT-PCR in roots and shoots of wild-type, *esb1-1* and *esb1-2* plants. RNA was isolated from shoot and root of 5 week-old plants grown in soil under short-day conditions. The expression of actin (At2g37620) was included in all calculations as an internal normalization standard across samples, and expression relative to actin calculated as 2∧^(ΔCT)^. Data represent means of 3 plants from each genotype, with 3 replicate real-time RT-PCR reactions per plant. Error bars represent the range around the mean calculated as 2∧^(ΔCT±SD(ΔCT))^ where SD(ΔCT) is calculated from composite SD of actin and At2g28670. B. Expression pattern of At2g28670 in root tissue of wild-type Col-0. Data derived from the Arabidopsis Gene Expression Database (http://arexdb.org/index.jsp) [21],[22].

Expression of At2g28670 in wild-type, *esb1-1* and *esb1-2* plants was quantified in root and shoot tissue using quantitative RT-PCR (Figure 4A). At2g28670 was strongly expressed in roots of wild-type plants with little or no expression in shoots. Expression of At2g28670 was lost in both *esb1-1* and *esb1-2*. Searching the Arabidopsis Gene Expression Database (AREX) [21],[22] for information on the expression pattern of At2g28670 revealed that expression of this gene is primarily in the root endodermis with lower levels of expression in the quiescent center, stele and cortex (Figure 4B).

**Figure 5:**
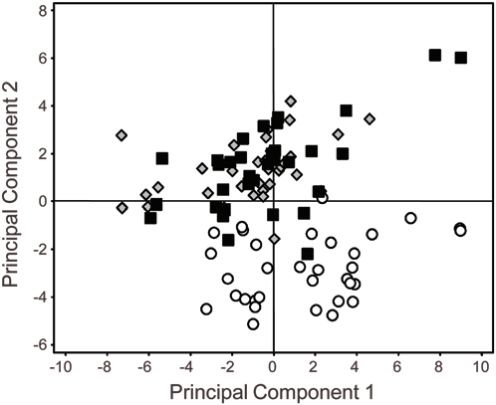
Principal component analysis of the shoot ionome of five week old wild-type Col-0, *esb1-1* and *esb1-2*. PCA based on the shoot concentrations of Li, B, Na, P, K, Ca, Mn, Fe, Co, Ni, Cu, Zn, As, Se, Mo and Cd in wild-type Col-0 (open circles), *esb1-1* (grey diamonds) and *esb1-2* (solid squares). The analysis was performed on data from n = 33 plants from each genotype. The raw data can be viewed and obtained from www.ionomicshub.org in trays 1095 and 1146.

To establish that loss-of-function of At2g28670 is driving the shoot ionomic phenotype observed in *esb1-1* we measured the elemental composition of leaf material from *esb1-1*, *esb1-2* and wild-type plants grown together. Using principal component analysis (PCA) of the leaf elemental composition of each genotype we determined that *esb1-1* and *esb1-2* plants clustered together and formed a single group that was distinct from wild-type Col-0 (Figure 5). Furthermore, *esb1-1* and *esb1-2* show the same changes in the elemental composition of leaf tissue compared to wild-type plants (Table 1).

### Loss-of-Function of At2g28670 Doubles the Aliphatic Monomer Content of Suberin

**Figure 6:**
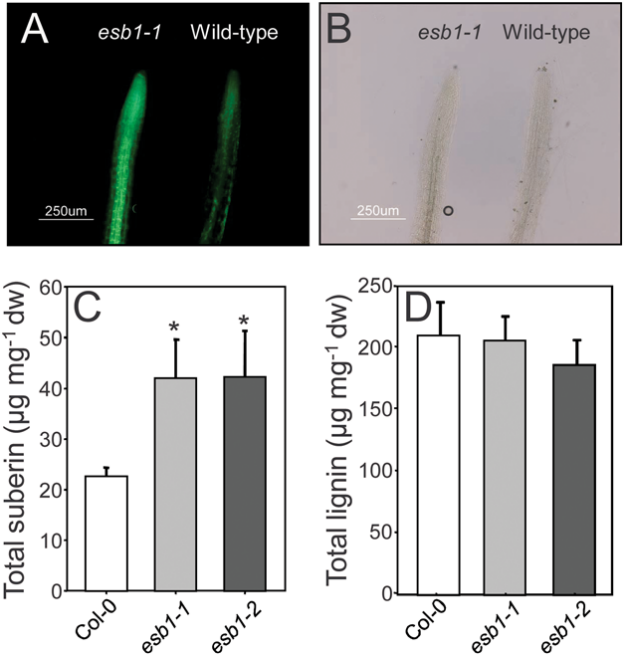
Total root suberin and lignin in five week old wild-type Col-0, *esb1-1* and *esb1-2*. A. Aliphatic components of suberin were visualized by ultraviolet illumination using a fluorescence microscope after staining root tissue with Fluorol Yellow. B. Bright field image of the same roots shown in (A). C. Total content of the aliphatic components of suberin in roots of five week old wild type and mutant plants. Data represents mean values in µg per mg dry weight±standard deviations of wild type (Col-0) (n = 7), *esb1-1* (n = 11) and *esb1-2* (n = 3). Each sample containing 4–5 roots for each genotype. D. Total root lignin content of five week old wild type and mutant plants. Data represents mean values in µg per mg dry weight±standard deviations of wild type (Col-0), *esb1-1* and *esb1-2* with n = 4 biological replicates for each genotype, with each sample containing 4–5 roots for each genotype. * indicates data that is significantly different from wild-type Col-0 (t-test P<0.01).

**Figure 7:**
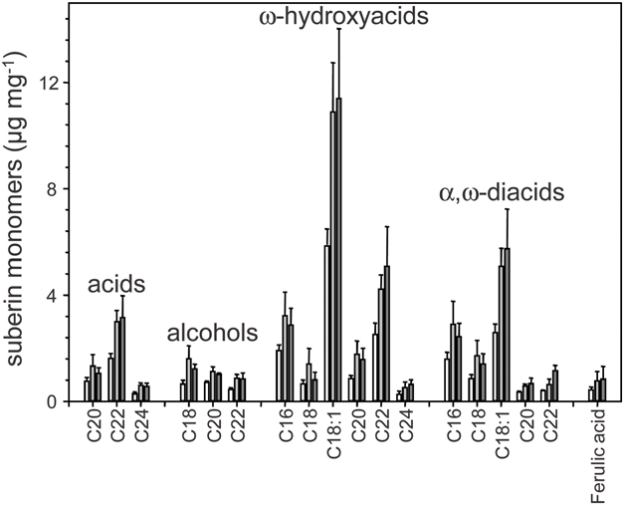
Suberin aliphatic monomer composition in roots of five week old wild-type and mutant plants. Suberin aliphatic monomers were analyzed using gas chromatography after release by transesterification using boron trifluoride in methanol from polysaccharide hydrolase digested and solvent extracted root cell walls. Absolute amounts of suberin monomers are shown as mean values in µg per mg dry weight±standard deviations of wild type (Col-0) (white bar; n = 7), *esb1-1* (light grey bar; n = 11) and *esb1-2* (dark grey bar; n = 3). Each sample containing 4–5 roots for each genotype.

Expression of At2g28670 is primarily in the root endodermis, the major site of suberin deposition in the primary root, and the localization of the Casparian strip. Staining of the aliphatic components of suberin using Fluoral Yellow revealed a clear increase in staining in roots from *esb1-1*, when compared to wild-type roots. In particular, increased staining is observed in the two rows of cells in the central portion of the root, suggesting increases in endodermal suberin (Figure 6A & B). Slightly elevated staining is also observed more diffusely in the root tip. Total aliphatic monomer content in both *esb1-1* and *esb1-2* was double that of wild-type roots (Figure 6C). However, there was no difference in total lignin content of roots of *esb1-1* or *esb1-2* (Figure 6D). Further analysis of the individual aliphatic monomer components of suberin revealed a doubling of all the acids, alcohols, ω-hydroxyacids, α,ω-diacids and ferulic acid components measured in both *esb1-1* and *esb1-2* compared to wild-type roots (Figure 7 and Supplemental Table 1).

### Role of the Root in Determining the Shoot Ionomic Phenotype of *Esb1*

**Figure 8:**
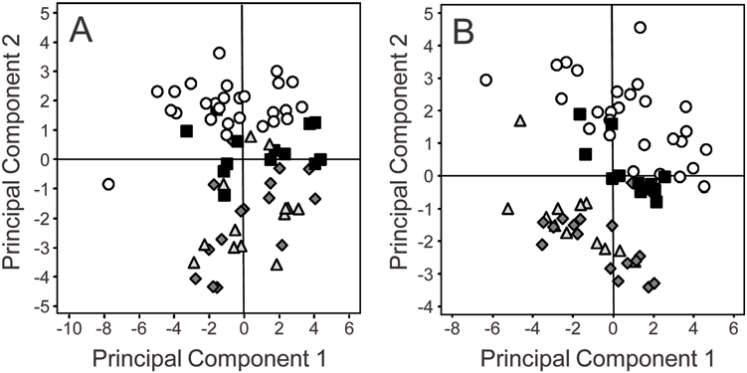
Principal component analysis of the shoot ionome of five week old wild-type Col-0 and *esb1* grafted plants. PCA based on the shoot concentrations of Li, B, Na, P, K, Ca, Mn, Fe, Co, Ni, Cu, Zn, As, Se, Mo and Cd. A. Wild-type Col-0 self grafted (open circles), *esb1-1* self grafted (grey diamonds), wild-type Col-0 shoot/*esb1-1* root grafted (grey triangles) and *esb1-1* shoot/wild-type Col-0 root grafted (solid squares). B. Wild-type Col-0 self grafted (open circles), *esb1-2* self graft (grey diamonds), wild-type Col-0 shoot/*esb1-2* root grafted (grey triangles) and *esb1-2* shoot/wild-type Col-0 root grafted (solid squares). The analysis was performed on data from n = 11–27 plants from each grafting type.

Given that At2g28670 is primarily expressed in the root, and that loss-of-function of this gene results in a doubling of root suberin and a corresponding alteration in the leaf ionome, we hypothesized that root processes are responsible for the leaf ionomic phenotype of *esb1*. To test this hypothesis we performed a reciprocal grafting experiment in which we grafted wild-type scions onto *esb1* rootstocks and *esb1* scions onto wild-type rootstocks. Grafting of wild-type scions onto *esb1* rootstocks (both *esb1-1* and *esb1-2*) revealed that the leaf ionomic phenotype of *esb1* is dependent on the rootstock. PCA of the leaf elemental composition indicated that plants with *esb1* and wild type roots clustered separately, regardless of scion (Figure 8A & B).

### Effect of Loss-of-Function of At2g28670 on Whole Plant Water Relations

**Figure 9:**
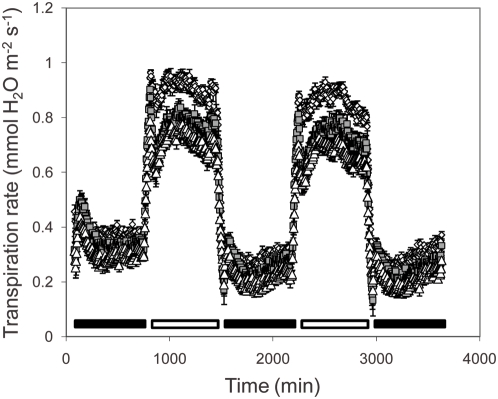
Transpiration rates of five week old wild-type and mutant plants. Five week old plants of Col-0, *esb1-1* and *esb1-2* grown under 12 hr/12 hr day/night were used for the transpiration experiment. Water loss from each plant was measured as weight change at 5 minute intervals over 62 hr. At the end of the experiment leaf area was measured and transpiration rate calculated. Data represents the mean±standard error of n = 6–7 replicate plants for each genotype. White diamonds = wild-type Col-0, grey squares = *esb1-1* and white triangles = *esb1-2*. Black horizontal bars represent nighttime period, white bars represent daytime period.

**Figure 10:**
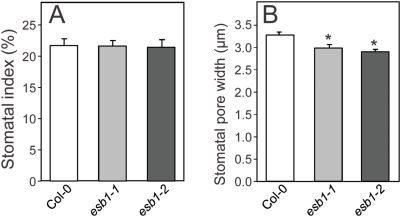
Stomatal index and stomatal aperture of five week old wild-type Col-0 and mutant plants. A. Stomatal index was calculated as the number of stomata as a percentage of the total cell number (epidermal cells+stomata) in a given leaf area. Measurement was performed on five week old plants grown with a 12 hr day length. Images were captured by scanning electron microscopy. Data represents the mean±standard error of the stomatal index calculated from the number of stomata and epidermal cells counted in a 0.18 mm^2^ area of 1–3 leaves from 2–4 independent plants for each genotype. On average 35 stomata and 130 epidermal cells were counted for each 0.18 mm^2^ area recorded. B. Stomatal aperture was measured using epidermal cell imprints on five week old plants. Data represents the mean±standard error of stomatal apertures measured from 1–2 leaves sampled from 8–11 independent plants for each genotype. Total stomatal apertures measured for wild-type Col-0 = 196, *esb1-1* = 136 and *esb1-2* = 203. * indicates data that is significantly different from wild-type Col-0 (t-test P<0.01).

Suberin has been proposed to form a barrier to water movement through the root apoplast. Elevated suberin, as found in *esb1*, should therefore enhance this barrier, reducing both water movement to the shoot and water loss from the root back to the soil. To test this hypothesis we measured both transpiration and wilting after water withdrawal in *esb1*. Peak daytime transpiration rates in *esb1-1* and *esb1-2* plants were approximately 15% of those of wild-type plants (Figure 9). The stomatal index (ratio of guard cells to epidermal pavement cells) was unaltered in *esb1* compared to wild-type (Figure 10A), establishing that the reduction in transpiration in *esb1* is not driven by a simple alteration in stomatal density. However, the stomatal pore width in *esb1* was reduced by approximately 10% compared to wild-type (P<0.01) (Figure 10B), and the magnitude of this change is consistent with the reduction in transpiration observed.

**Figure 11:**
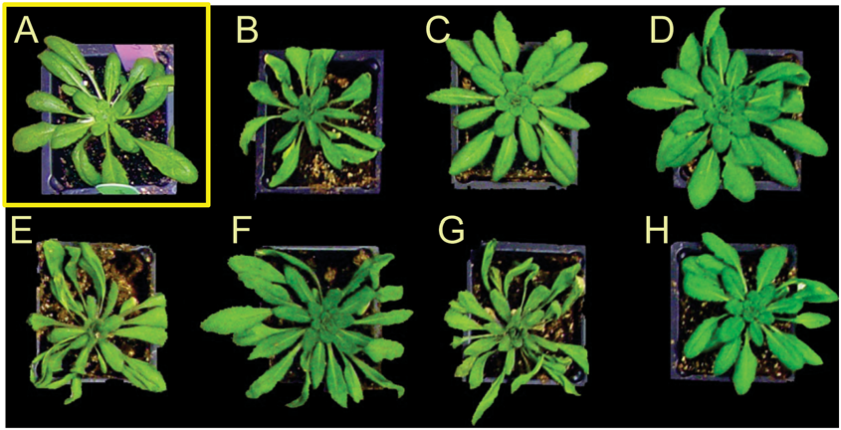
Wilting resistance of grafted wild-type Col-0 and mutant plants. After grafting plants were grown for 3 weeks in soil with regular watering, after which time watering was stopped and the plants' wilting status recorded at 11 days after water withdrawal. A. Wild-type Col-0 without water withdrawal as a control. B. Self grafted wild-type Col-0. C. Self grafted *esb1-1*. D. Self grafted *esb1-2*. E. *esb1-1* shoot/wild-type Col-0 root grafted. F. Wild-type Col-0 shoot/*esb1-1* root grafted. G. *esb1-2* shoot/wild-type Col-0 root grafted. H. Wild-type Col-0 shoot/*esb1-2* root grafted.

**Figure 12:**
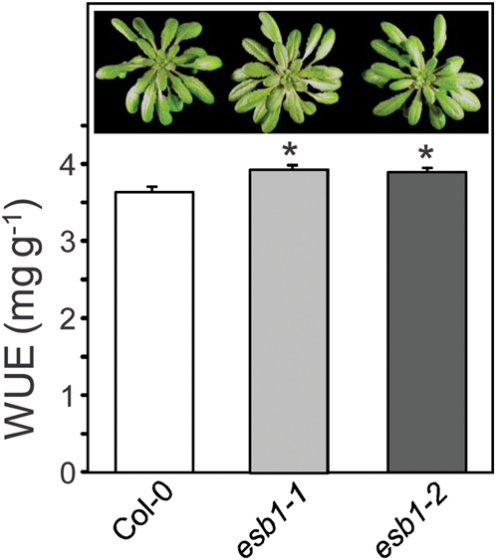
Water use efficiency of wild-type Col-0 and mutant plants. Water use efficiency is the amount of water loss calculated per unit dry weight of plants. The amount of water used by each plant was measured over a five week period (34 days) using plants grown under 12 hr/12 hr day/night. At the end of five weeks shoot dry weight was measured. Data represents the mean±standard error of n = 30–35 independent plants for each genotype. * indicates data that is significantly different from wild-type Col-0 (t-test P<0.01). Insert. Example of plants with the three different genotypes after 34 days' growth at the end of the experiment.

To test the rate of wilting in *esb1*, watering was stopped after 5-weeks of growth and the plants were monitored for wilting. Both *esb1-1* and *esb1-2* showed reduced wilting after water withdrawal compared to the wild type plants (Figure 11). Using reciprocal grafting experiments we further determined that this enhanced wilting resistance is determined by the root. Plants with *esb1* rootstock (both *esb1-1* and *esb1-2*) grafted to wild-type scions showed reduced wilting similar to *esb1* self-grafted plants, whereas plants with wild-type rootstock all wilted within 11 days (Figure 11). Water use efficiency (WUE) of a plant relates water transpired to biomass produced, and is an important parameter for the assessment of the impact of reductions in transpiration on plant productivity. We have established that elevated suberin in *esb1* roots is related to a decrease in transpiration and increased water stress tolerance. These reductions in root-controlled water loss in *esb1* cause a small (∼14%) reduction in dry biomass production, but an overall increased in the amount of biomass produced per unit of water transpired, with both *esb1-1* and *esb1-2* showing small (8%) but significant (P<0.01) increases in WUE (Figure 12).

## Discussion

Radial rather than axial transport is known to limit water loading by roots [23]. The force driving water across the root is provided by tension (negative pressure) created by transpiration, and flow is thought to be primarily apoplastic. However, during periods of reduced transpiration, at night or in dry or saline soils, it is root pressure, caused by osmotic water flow driven by the uptake of nutrients into the xylem that drives water transport to the shoots. This osmotic pathway for water is primarily cell-to-cell. It has been proposed that this “Composite Model” for water uptake allows plant roots to take up water under various conditions and balance it with demands [24]. However, because the Casparian strip is thought to be somewhat permeable to water, but not to mineral ions, transport of water and solutes are differentiated in the endodermal apoplast, with the Casparian strip forming the main barrier to radial ion movement [25]. However, to date no mutants with elevated root suberin have been described to help elucidate this model further.

The *esb1* mutant described here has significantly higher root suberin compared to wild type plants, and grafting experiments establish that this increase in suberin is responsible for the observed changes in the shoot ionome. The final accumulation of mineral elements in the shoot would be expected to be a balance between the negative effect of suberin reducing radial apoplastic solute transport, and the positive effect of the reduced transpiration of *esb1* on the xylem concentration of solutes. The reductions in total shoot Ca, Mn and Zn observed in *esb1* suggest that a significant component of the radial root transport of these elements is via the apoplast. This evidence confirms the earlier conclusion of White [26], based on modeling of physiological and biochemical parameters, that a significant root apoplastic bypass pathway exists for Ca. It is also consistent with the observation that increased endodermal cytoplasmic Ca during cooling is inversely proportional to the level of suberin deposition [27]. Further, the conclusion by White and coworkers [28] that Zn may also reach the xylem via an apoplastic pathway is also supported by our data on *esb1*.

The increase in shoot S, K, As, Se and Mo in *esb1* suggests that these elements may be primarily transported radially in the root via a cell-to-cell pathway, making them resistant to changes in endodermal suberin, and more prone to the influence of increasing xylem concentrations of solutes due to the reduced transpiration driven water flow in *esb1*. We also observe Na concentration increasing in the shoot of *esb1*, suggesting the absence of an apoplastic bypass pathway for this element at the low external Na concentrations present in the potting mix used in these studies. However, elevated suberin has been implicated in reducing Na accumulation and increasing Na tolerance in rice [29],[30]. Such evidence suggests that at low external Na apoplastic bypass flow is limited. However, at elevated external Na concentrations apoplastic bypass flow becomes significant, and reduction in this flow can lead to elevated Na tolerance.

It has previously been reported that Ca and Mg shoot concentrations are positively correlated across taxa [31], within-species and genetically [32]. We also observe a strong correlation between Ca and Mg in both wild-type and *esb1* (R^2^ = 0.75 and 0.68, respectively. Data taken from trays 533, 534 and 535, n = 30 for each genotype; see www.ionomicshub.org). Such correlations suggest that the mechanisms driving Ca and Mg accumulation are related. However, the fact that in *esb1* Ca is reduced without significantly changing Mg suggests that the regulatory mechanisms controlling Ca and Mg can be separated.

Elevated root suberin in *esb1* is also associated with a root-dependent increase in time to wilting during water stress. Resistance to wilting can be achieved via a reduction in water loss to the environment, from both the root and leaves, or through an increase in the ability to take up water from the soil. Delayed wilting in *esb1* appears to be related to the reduced stomatal aperture and reduced daytime transpiration rates in this mutant. However, grafting experiments establish that this delayed wilting in *esb1* is a root-dependent phenomenon, suggesting signaling from root to shoot. A possible explanation for this signal is that roots of *esb1* are constitutively responding to water stress, due to increased suberin causing enhanced hydraulic resistance to radial transport of water. This stress is transmitted to the shoot via ABA, or directly as a hydraulic signal [33]. Such a model remains to be tested.

Reduced transpiration is often correlated with reduced biomass accumulation, as observed here for *esb1*. However, biomass reduction in *esb1* is less than the reduction in transpiration, leading to an overall increase in water use efficiency of *esb1*. Increased root suberin may therefore present new opportunities for developing enhancing drought resistance in crop plants.

Here we establish that loss-of-function of the endodermally expressed gene At2g28670 leads to a doubling of all the aliphatic components of root suberin. The enzymatic function of the protein encoded by At2g28670, and how this function affects suberin, remains an open question. Importantly, the elevated root suberin observed in two independent At2g28670 loss-of-function mutants (*esb1-1* and *esb1-2*) directly affects the shoot ionome, causing several changes, including a 50% reduction in Ca and a 40% increase in Na. These changes are also associated with decreased transpiration and increased wilting resistance.

Overall, these observations provide strong experimental support for the standard model that suberin acts as an extracellular transport barrier limiting apoplastic radial transport of water and solutes. Our observations suggest that elevation of root suberin may represent another approach to the development of drought resistant crops with improved WUE. Furthermore, manipulation of suberin may also provide new opportunities for the development of plant-based foods with altered mineral nutrient contents.

## Materials and Methods

### Plant Material and Plant Growth Conditions

All *A. thaliana* lines were obtained from the ABRC or Lehle seeds. All T-DNA lines analyzed were homozygous for the T-DNA insertion. Unless stated otherwise all plants were grown in a controlled environment, 8 h light∶16 h dark (90 µmol m^−2^ s^−1^ photosynthetically active light) and 19 to 22°C as previously describe [14]. Briefly, seeds were sown onto moist soil (Sunshine Mix LB2; Carl Brehob & Son, Indianapolis, Indiana, United States) with various elements added at subtoxic concentrations (As, Cd, Co, Li, Ni, Pb, and Se [14]) and stratified at 4°C for 3 d. Plants were bottom-watered twice per week with 0.25× Hoagland solution in which iron was replaced with 10 µM Fe-HBED [N,N′-di(2-hydroxybenzyl)ethylenediamine-N,N′-diacetic acid monohydrochloride hydrate; Strem Chemicals, Inc., http://www.strem.com]. For elemental analysis after 5-weeks, plants were nondestructively sampled by removing one or two leaves. The plant material was rinsed with 18 MΩ water and placed into Pyrex digestion tubes.

### Tissue Elemental Analysis

Tissue samples were dried at 92°C for 20 h in Pyrex tubes (16×100 mm) to yield approximately 2–4 mg of tissue for elemental analysis. After cooling, seven of approximately 100 samples from each sample set were weighed. All samples were digested with 0.7 ml of concentrated nitric acid (OmniTrace; VWR Scientific Products; http://www.vwr.com), and diluted to 6.0 ml with 18 MΩ water. Elemental analysis was performed with an ICP-MS (Elan DRCe; PerkinElmer, http://www.perkinelmer.com) for Li, B, Na, Mg, P, S,K, Ca, Mn, Fe, Co, Ni, Cu, Zn, As, Se, Mo, and Cd. All samples were normalized to calculated weights, as determined with an iterative algorithm using the best-measured elements, the weights of the seven weighed samples, and the solution concentrations, described in [14], and implemented in the PiiMS database [34]. An Excel implementation of this algorithm is available at www.ionomicshub.org along with validation data sets.

### DNA Microarray-Based Bulk Segregant Analysis and Deletion Mapping

DNA microarray-based BSA was performed as previously described [17],[35]. Briefly, SFPs were identified between Col-0 and L*er*-0 by hybridizing labeled genomic DNA from each one of the accessions to Affymetrix ATH1 microarrays and comparing them to Col-0 hybridizations downloaded from http://www.naturalvariation.org/xam. Two genomic DNA pools from an F2 population of a cross between L*er*-0 and the *esb1-1* mutant in the Col-0 background were created and hybridized to separate DNA microarrays. Each one of the pools contained plants with either shoot Ca and B contents similar to Col-0 (“control” pool) or low shoot Ca and B contents similar to *esb1-1* (“low Ca and B” pool). At loci unlinked to the low Ca and B phenotype, the pools should have equivalent amounts of each genotype, and the hybridization signal at each SFP should be intermediate between the two parent accessions, for an average difference between the two DNA microarrays of zero. At linked loci, the difference between the two DNA pools should be approximately two-thirds the difference between the parent accessions. By smoothing the signal across multiple SFPs, noise is reduced and the peak of the differences in hybridization signal will correspond to the chromosomal region of the loci controlling the low Ca and B trait. Raw hybridization data (.CEL files) for each probe on the ATH1 DNA microarrays used in these experiments have been submitted to the Gene Expression Omnibus and are accessible through GEO Series accession number GSE15655 (http://www.ncbi.nlm.nih.gov/geo/query/acc.cgi?acc=GSE15655). For the deletion mapping, DNA was extracted from Col-0 and *esb1-1* plants and hybridized to the ATTILE 1.0R array using the same protocols as described above. After quantile normalization, the difference in hybridization intensity for each probe between 9 and 13 Mb was visually inspected to identify the causal locus.

### Grafting of Arabidopsis

Seedlings were grafted as previously described [16]. Plants were harvested for shoot elemental analysis 4 weeks after transfer to soil. Postharvest analysis of graft unions was performed under the stereoscope to identify any adventitious root formation from grafted individuals. Individuals with adventitious roots emerging at or above the graft union or without a clear graft union were eliminated from subsequent analyses.

### Quantitative Real-Time RT-PCR

Plants were first analyzed by ICP-MS and further used to determine *esb1* transcript levels as described previously [16]. For *esb1* (At2g28670) transcript quantification the following primers were used: forward primer 5′-ATGTCCCTTTCCTCGTTGGA-3′ and reverse primer 5′-GCCACTAGCAACAGGGAAACC-3′. Three reactions were done per biological sample and three independent replicate samples per genotype were used to evaluate the transcript abundance of *esb1*. Data was analyzed using the SDS software (Applied Biosystems version 1.0), following the method of Livak and Schmittgen [36]. CT values were determined based on efficiency of amplification. The mean CT values were normalized against the corresponding ACTIN 1 gene (At2g37620) and CT values calculated as (CT *esb1*- CT *Actin1*). The expression of *esb1* was calculated using the 2∧^( ΔCT)^ method. The final error was estimated by evaluating the 2∧^( ΔCT)^ term using 2∧^( ΔCT)^ plus standard deviation and 2∧^( ΔCT)^ minus the standard deviation [36].

### Suberin Analysis

*In vivo* staining of suberin was performed using Fluorol Yellow, following the method of [37]. Roots of 1 week old seedlings, grown on 1/2 MS agar plates, were incubated in a freshly prepared 0.01%(w/v) solution of Fluorol Yellow 088 (Sigma) in lactic acid at 70°C for 1 h. Parallel stained roots of Col-0 and *esb1-1* mutant plants were placed adjacent on a microscope slide and observed under UV-light using an Axioplan microscope (Zeiss, Germany). For quantitative chemical suberin analysis [38] roots of 35 d old soil-grown plants were incubated in 1% (v/v) cellulase (Celluclast, Novozymes, Germany), 1% (v/v) pectinase (SIHA, Novozymes, Germany) in 10 mM citric buffer pH 3 containing 10 mM NaN_3_. After 10 d the root cell wall material was washed and incubated in 10 mM sodium tetraborate (pH 9.0) for 2 d and extracted with chloroform∶methanol (1∶1, v/v) to remove unbound lipids. The cell wall material of 4–5 plants was depolymerized by transesterification in 1 ml borontrifluorid in methanol (10%, Fluka) for 16 h at 70°C. After adding 10 µg dotriacontane (internal standard) the methanolysate was transferred into 2 ml saturated NaHCO_3_/H_2_O and suberin monomers were subsequently extracted in chloroform. Free hydroxyl and carboxyl groups were derivatized with bis-(N,O-trimethylsilyl)-tri-fluoroacetamide (BSTFA, Macherey-Nagel, Germany) (20 µl BSTFA+20 µl Pyridin, 40 min, 70°C) prior to GC–MS/FID analysis. Suberin monomers were injected on-column (DB-1 (J&W Scientific), 30 m×0.32 mm, 0.1 µm), separated and identified on an Agilent 6890N gas chromatograph coupled with an Agilent 5973N quadrupole mass selective detector (70 eV, m/z 50–700). The following temperature gradient was applied: 2 min at 50°C, 10°C/min to 150°C, 1 min at 150°C, 3°C/min to 310°C, 15 min at 310°C. Quantitative determination of the components was carried out with an identical GC-system coupled with a flame ionisation detector based on the internal standard.

### Lignin Quantification

Lignin content in roots was determined by the acetyl bromide method using 3–5 mg root material as used for suberin analysis. 1 ml acetyl bromide/acetic acid (1∶3) was added to the root cell wall material and incubated at 70°C for 30 min. After cooling to 15°C 0.9 ml 2 M NaOH and 5 ml acetic acid was added. Finally 0.1 ml 7.5 M hydroxylamine-HCl and acetic acid was added to a final volume of 10 ml. The lignin content was calculated from the A_280_ using the extinction coefficient 24 g^−1^ L cm^−1^.

### Analysis of Transpiration Rates

Plants were grown for 5 weeks in 2 inch pots with 12 hr of photosynthetically active light (80–100 µmol/m^2^/s), with mean day and night temperatures of 22 and 18°C, respectively. For analysis of transpiration rates, pots were covered with plastic wrap (Saran wrap) to avoid water loss from the soil, and placed on one of twenty balances (EK-410i, A&D) to monitor changes in weight. Weights of pots were automatically recorded using balances connected to computers through WinWedge software (TAL technologies Inc.) at 5 min intervals for 2 days and 3 nights. A total of six to seven plants per genotype were analyzed. At the end of the experiment total leaf area for each plant was determined by digitally recording images of all leaves and using ImageJ [39] to determine leaf area.

### Analysis of Stomatal Density and Aperture

Five week old plants grown under the conditions described above for measurement of transpiration were used for measuring stomatal index and aperture. To determine stomatal index, stomata were observed on the abaxial surface of leaves using a scanning electron microscope (JSM-840, JEOL) at 250× magnification. Stomatal index was calculated as the ratio of the number of stomata to the total number of cells (epidermal cells and stomata) in an area of 0.18 mm^2^. To determine stomatal aperture we modified the protocol of Hilu and Randall [40]. Briefly, clear nail polish was applied to the abaxial surface of leaves, peeled when dry and stomatal impression in the nail polish observed under a light microscope (Vanox-S, Olympus) at 400× magnification and images recorded digitally. Stomatal aperture width was measured using ImageJ software [39].

### Wilting Resistance

Grafting was done as described above. To measure wilting resistance grafted plants were transferred to soil and grown for three weeks, after which watering was stopped and plants observed for symptoms of wilting for 11 days.

### Water-Use Efficiency

Plants were grown in inverted brown 50 mL Falcon tubes (Greiner Bio-One) filled with soil (1∶1 proportion of Premier ProMix PGX and calcined clay (Turface MVP)) and covered with a screw cap with a mesh insert to allow for water uptake. A 4–5 mm diameter hole was made on the narrow end of the Falcon tube for seed germination and growth. Plants were grown in a growth chamber with 10 hr of photosynthetically active light (80–100 µmol/m^2^/s), with mean day and night temperatures of 22 and 18°C, respectively. Watering was done with alternating clean or fertilized (Miracle Gro Excel, Scotts) water. To monitor water use tubes containing plants, and also control tubes with no plants, were weighted (Balance- GH-252, A&D) before and after each watering, over a period of five weeks. At the end of the experiment whole shoots were harvested for each plant, dried at 70°C and the dry weight determined. Water use efficiency was calculated by dividing total shoot dry weight by the amount of water utilized by the plants over the complete growth cycle.

## Supporting Information

Table S1 Comparison of the suberin monomer composition in roots of wild type and *esb1* plants. Suberin aliphatic monomers were analyzed using gas chromatography. Absolute amounts of suberin monomers are shown as mean values in µg per mg dry weight±standard deviations. Means were calculated from biologcal replicates (Col-0 n = 7, *esb1-1* n = 11 and *esb1-2* n = 3) with 4–5 roots per genotype for each sample.
(0.10 MB DOC)
