# Supplementary material for: Root Suberin Forms an Extracellular Barrier That Affects Water Relations and Mineral Nutrition in Arabidopsis
Source: PLoS Genet. 2009 May 22;5(5):e1000492. doi: 10.1371/journal.pgen.1000492 (PMC2679201; doi:10.1371/journal.pgen.1000492)
Supplement: Table S1 — Comparison of the suberin monomer composition in roots of wild type and esb1 plants. Suberin aliphatic monomers were analyzed using gas chromatography. Absolute amounts of suberin monomers are shown as mean values in µg per mg dry weight±standard deviations. Means were calculated from biologcal replicates (Col-0 n = 7, esb1-1 n = 11 and esb1-2 n = 3) with 4–5 roots per genotype for each sample. (0.10 MB DOC) [file pgen.1000492.s001.doc]

**Supplemental Table 1: Comparison of the suberin monomer composition in roots of wild type and *esb1* plants**

|  | **Col-0** | |  | ***esb1-1*** | | | |  | ***esb1-2*** | | | |
| --- | --- | --- | --- | --- | --- | --- | --- | --- | --- | --- | --- | --- |
|  | **[µg/mg]** | |  | **[µg/mg]** | |  |  |  | **[µg/mg]** | |  |  |
| **Suberin monomer (chain length)** | **mean** | **sd** |  | **mean** | **sd** | **P-value** | **change vs. WT** |  | **mean** | **sd** | **P-value** | **change vs. WT** |
| **Fatty acids** |  |  |  |  |  |  |  |  |  |  |  |  |
| Eicosanoic acid (C20) | 0,76 | 0,14 |  | 1,33 | 0,43 | P < 0.01 | ↑ |  | 1,04 | 0,22 | 0,04 | ↑ |
| Docosanoic acid (C22) | 1,62 | 0,19 |  | 3,00 | 0,43 | P < 0.01 | ↑ |  | 3,16 | 0,83 | P < 0.01 | ↑ |
| Tetracosanoic acid (C24) | 0,28 | 0,07 |  | 0,60 | 0,09 | P < 0.01 | ↑ |  | 0,55 | 0,13 | P < 0.01 | ↑ |
|  |  |  |  |  |  |  |  |  |  |  |  |  |
| **Alcohols** |  |  |  |  |  |  |  |  |  |  |  |  |
| Octadecanol (C18) | 0,65 | 0,14 |  | 1,60 | 0,49 | P < 0.01 | ↑ |  | 1,22 | 0,17 | P < 0.01 | ↑ |
| Eicosanol (C20) | 0,71 | 0,05 |  | 1,13 | 0,18 | P < 0.01 | ↑ |  | 1,02 | 0,05 | P < 0.01 | ↑ |
| Docosanol (C22) | 0,44 | 0,06 |  | 0,87 | 0,15 | P < 0.01 | ↑ |  | 0,84 | 0,22 | P < 0.01 | ↑ |
|  |  |  |  |  |  |  |  |  |  |  |  |  |
| **ω-Hydroxyacids** |  |  |  |  |  |  |  |  |  |  |  |  |
| 16-Hydroxy-hexadecanoic acid (C16) | 1,91 | 0,22 |  | 3,22 | 0,90 | P < 0.01 | ↑ |  | 2,87 | 0,64 | P < 0.01 | ↑ |
| 18-Hydroxy-octadecanoic acid (C18) | 0,66 | 0,15 |  | 1,39 | 0,60 | P < 0.01 | ↑ |  | 0,80 | 0,29 | 0,30 | ↑ |
| 18-Hydroxy-octadecenoic acid (C18 (1)) | 5,84 | 0,60 |  | 10,89 | 1,86 | P < 0.01 | ↑ |  | 11,40 | 2,63 | P < 0.01 | ↑ |
| 20-Hydroxy-eicosanoic acid (C20) | 0,85 | 0,13 |  | 1,77 | 0,51 | P < 0.01 | ↑ |  | 1,57 | 0,43 | P < 0.01 | ↑ |
| 22-Hydroxy-docosanoic acid (C22) | 2,51 | 0,44 |  | 4,22 | 0,55 | P < 0.01 | ↑ |  | 5,08 | 1,48 | P < 0.01 | ↑ |
| 24-Hydroxy-tetracosanoic acid (C24) | 0,26 | 0,13 |  | 0,51 | 0,21 | 0,01 | ↑ |  | 0,64 | 0,17 | P < 0.01 | ↑ |
|  |  |  |  |  |  |  |  |  |  |  |  |  |
| **α,ω-Dicarboxylic acids** |  |  |  |  |  |  |  |  |  |  |  |  |
| Hexadecane-1,16-dioic acid (C16) | 1,59 | 0,26 |  | 2,90 | 0,87 | P < 0.01 | ↑ |  | 2,44 | 0,51 | P < 0.01 | ↑ |
| Octadecane-1,18-dioic acid (C18) | 0,85 | 0,15 |  | 1,72 | 0,58 | P < 0.01 | ↑ |  | 1,40 | 0,40 | 0,010 | ↑ |
| Octadecene-1,18-dioic acid (C18 (1)) | 2,59 | 0,32 |  | 5,08 | 0,67 | P < 0.01 | ↑ |  | 5,73 | 1,50 | P < 0.01 | ↑ |
| Eicosane-1,20-dioic acid (C20) | 0,34 | 0,05 |  | 0,56 | 0,08 | P < 0.01 | ↑ |  | 0,66 | 0,22 | P < 0.01 | ↑ |
| Docosane-1,22-dioic acid (C22) | 0,41 | 0,03 |  | 0,62 | 0,22 | 0,02 | ↑ |  | 1,15 | 0,21 | P < 0.01 | ↑ |
|  |  |  |  |  |  |  |  |  |  |  |  |  |
| Ferulic acid | 0,42 | 0,12 |  | 0,76 | 0,37 | 0,03 | ↑ |  | 0,84 | 0,48 | 0,05 | ↑ |

Suberin aliphatic monomers were analyzed using gas chromatrogaphy. Absolute amounts of suberin monomers are shown as mean values in µg per mg dry weight ± standard deviations. Means were calculated from biologcal replicates ( Col-0 n= 7, *esb1-1* n = 11 and *esb1-2* n = 3) with 4 - 5 roots per genotype for each sample.
